# Topical Dopamine Application on Form-Deprivation Myopia in Rabbits

**DOI:** 10.3390/life15030461

**Published:** 2025-03-14

**Authors:** Dong Hyun Kim, Jeong-Min Hwang, Hee Kyung Yang

**Affiliations:** Department of Ophthalmology, College of Medicine, Seoul National University, Seoul National University Bundang Hospital, Seongnam 13620, Republic of Korea; himrdh@gmail.com (D.H.K.); hjm@snu.ac.kr (J.-M.H.)

**Keywords:** topical dopamine, rabbit myopia, form deprivation myopia

## Abstract

**Background/Objectives**: This study aimed to investigate the efficacy of topical dopamine administration in inhibiting form deprivation (FD) myopia in a rabbit model. **Methods**: A total of 16 neonatal New Zealand white rabbits were randomly assigned to two groups: a control group and a dopamine treatment group. FD myopia was induced in both groups by applying a light diffuser to one eye. The dopamine group received daily topical instillations of 4% dopamine in the eye with FD myopia, while the control group received normal saline instillations over a four-week period. Axial length measurements were taken to assess the degree of myopia, and histological analysis was performed to evaluate retinal safety and structural integrity. **Results**: The results indicated that dopamine treatment significantly inhibited axial elongation of the FD eyes compared to the control group, with measurements of 15.07 ± 0.34 mm for the dopamine group versus 15.63 ± 0.33 mm for the control group (*p* = 0.015). Histological analysis showed no evidence of structural alterations or apoptosis in the retina, confirming the safety of topical dopamine. **Conclusions**: Topical dopamine appears to be a promising therapeutic approach for controlling the progression of myopia in a rabbit model, demonstrating significant efficacy in reducing axial elongation without inducing ocular toxicity. These findings highlight the potential of dopamine in managing myopia and warrant further investigation in clinical settings.

## 1. Introduction

Myopia affects a substantial portion of the population, with prevalence rates reaching 60% in Asia and 40% in Europe among school children [1]. As the prevalence of myopia increases, so does the risk of ocular pathologies such as glaucoma, retinal detachment and macular degeneration [2,3,4,5]. Consequently, myopia can lead to significant visual impairment and a heightened risk of severe ocular complications [6,7]. To address this growing public health concern, it is necessary to implement effective control managements for myopia progression.

As our knowledge of the pathophysiology of myopia has grown, a number of retinal signaling factors such as dopamine [8,9], acetylcholine [10], retinoic acid [11], nitric oxide [12] and glucagon [13] have been identified as potential contributors to the development of myopia. Dopamine, one of the most studied neurotransmitters in animal models of myopia, has been associated with a potent control of emmetropization [14,15]. Subsequent data from multiple experiments in different species suggest that dopamine acts as a “stop” signal in axial eye growth, with a decrease in dopamine levels in response to form-deprivation (FD), which then recover when the diffuser is removed [14,16,17,18,19]. Activation of retinal dopamine D1 receptor has been shown to suppress myopia development in various animal models, including mice and chicks, while antagonists enhanced it [20,21]. The synthesis and release of dopamine in response to light stimuli, along with the recent epidemiological evidence indicating a correlation between outdoor activity and the onset of myopia, lends support to the hypothesis that dopamine plays a role in regulating refractive eye growth [22,23,24,25,26,27,28].

Experimental studies have been conducted in various species to investigate the potential of increasing dopamine levels or enhancing dopamine receptor activity to inhibit myopic growth in FD myopia models [16,19,29,30,31,32]. Nevertheless, no studies have been conducted on the topical instillation of dopamine in FD myopia models with a light diffuser in mammals. Topical instillation studies have been conducted in chicks, yet differences in avian anatomy, such as the presence of cartilaginous layers in the sclera, necessitate further investigation in mammals [33,34]. In rabbits, intravitreal dopamine injections were studied in a lid-suture FD model [32]. However, lid-suture FD myopia may result in corneal flattening due to mechanical interactions between the eyelids and the cornea [35].

In this study, we developed a rabbit FD myopia model induced by a removable light diffuser and investigated whether topical dopamine instillation has an inhibitory effect on the development of FD myopia. We also evaluated the safety of repeated topical dopamine instillation via histological analysis.

## 2. Materials and Methods

### 2.1. Animals

The study protocol was approved and monitored by the Institutional Animal Care and Utilization Committee of Seoul National University Bundang Hospital (BA-1902-266-012-07) and adhered to the ARVO Statement for the Use of Animals in Ophthalmic and Vision Research. All methods were conducted in strict accordance with the relevant guidelines and regulations. Power calculations were not performed to determine group sizes but were based on previous experiments with similar designs. Animals were randomly assigned to treatment groups and investigators were masked in treatment conditions. All experiments of this study comply with the ARRIVE guidelines.

A total of 16 neonatal New Zealand white rabbits were obtained from the Ji Seok-young Medical Life Research Institute and were fed by their maternal rabbits. The rabbit kits were weaned from their mothers at 4 weeks of age and subsequently raised in independent cages. The rabbits were housed in individual cages within a room that was maintained at a constant temperature (21 °C). The lighting was adjusted to come on at 6 a.m. and go off at 6 p.m., thereby providing the rabbits with a daily cycle of 12 h of light and 12 h of darkness. Food and water were made available ad libitum, and a shelter was provided in the cage.

### 2.2. Form-Deprivation Myopia Induction

**Figure 1:**
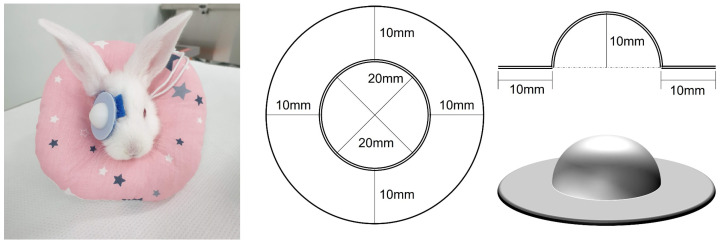
Detachable diffuser inducing form-deprivation myopia. A monocular detachable diffuser was attached to induce form-deprivation myopia in a rabbit eye (**Left**). 3D printer section plane drawing of the detachable diffuser (**Right**).

The eyes of the rabbit kits first opened at approximately 12 days of age. At 14 days of age, rabbits confirmed to be eyes open were fitted with a removable opaque diffuser over the right eye and worn for 4 weeks. The diffuser design and application protocol were adapted from those used in the guinea pig study [36]. The diffuser was 3D printed using polylactic acid, a bioplastic material. The diffuser comprised a semicircular dome and a ring-shaped hook-and-loop fastener, and another hook-and-loop fastener was placed in a ring around the rabbit’s eyes and attached with cyanoacrylate adhesive. This fastener was additionally fixed with 5-0 prolene (Figure 1).

At the Ji Seok-young Medical Life Research Institute, where the experimental animals are housed, full-time professional veterinarians are on duty and conduct regular ward rounds. A veterinarian made daily rounds, a cage keeper checked the cages at least twice a day, and researchers visited twice a day. In the unlikely event that the diffuser became dislodged, the researchers were able to almost immediately reattach it to maintain its stability. The diffusers were secured with cyanoacrylate adhesive and additional prolene sutures, which were protected by the wearing of a neck collar. When the cyanoacrylate adhesive was used alone, there was a single case of diffuser detachment in two controls, each at week 1, but, with the addition of prolene suture reinforcement and the wearing of a neck collar, there was no detachment at all.

### 2.3. Topical Dopamine Treatment

The subjects were randomly assigned to two groups of eight rabbits each: a vehicle control group and a dopamine group. In the dopamine group, one drop of dopamine HCl at 40 mg/mL (4% dopamine, Inopan; Myungmoon Pharm., Seoul, Republic of Korea) was instilled daily into the right FD eye, starting one week after applying the diffuser and continuing for the remaining four weeks of treatment. The right FD eyes of the control group received daily applications of 0.9% normal saline. The contralateral left eyes of both groups were maintained in a natural state, without the use of diffusers or eye drops. The male–female ratio of rabbits was 4:4 in the dopamine group and 3:5 in the control group. Gender was not considered a confounding factor because there are no known sex differences in the ocular growth response in rabbits [37].

### 2.4. Main Outcome Measures

Ophthalmic measurements were conducted following the administration of local anesthetic proparacaine hydrochloride 0.5% (Paracaine; Hanmi Pharmaceutical, Seoul, Republic of Korea). The anterior segment, including the cornea and lens, was examined on a weekly basis with a handheld slit lamp. Fluorescein strips were used for slit lamp examination. Conjunctival hyperemia, corneal epithelial defects or infiltrations and lens opacities were evaluated. Axial length was measured using high-frequency A-scan ultrasound at a frequency of 10 MHz (A-Scan Plus Connect; Accutome, Malvern, PA, USA). Axial lengths were measured five times in succession, and the mean values were recorded. The axial lengths of treated eyes and control eyes were compared, and the measurements were also compared between groups to verify the effect of topical dopamine. Slit lamp examinations and axial lengths were recorded weekly beginning at three weeks of age.

### 2.5. Histology

Following a four-week measurement period, the rabbits were sacrificed. To confirm the safety of the dopamine instillation procedure, the eyes of the sacrificed rabbits were enucleated for histological examination. In brief, the animals were anesthetized via intravenous injection of alfaxalone 6 mg/kg and xylazine 5 mg/kg. After the administration of anesthesia, a double dose of alfaxalone and xylazine was utilized to induce deeper anesthesia, after which the animals were sacrificed via intravenous injection of potassium chloride. Following sacrifice, the eyes were enucleated. The enucleated eye was fixed in 10% formalin and prepared for paraffin embedding, and 3-μm thick sections were cut. To investigate the impact of dopamine on retinal structure and retinal cells, a histology procedure with hematoxylin and eosin (H&E) staining was conducted in accordance with established protocols.

The TdT-mediated dUTP Nick-End labeling (TUNEL) assay was employed to detect apoptosis in the retina. TUNEL staining was conducted using a commercially available in situ apoptosis detection kit (ab206386; Abcam, Cambridge, UK) in accordance with a protocol adapted from the previous study [38] and the manufacturer’s instructions. Briefly, paraffin sections were deparaffinized in xylene and rehydrated in a graded alcohol series prior to treatment with proteinase K. 3% H_2_O_2_ was then added to inactivate the endogenous peroxidases. Apoptotic cells were labeled with terminal deoxynucleotidyl transferase, which catalyzes the addition of biotin-labeled deoxynucleotides, followed by incubation with a streptavidin-horseradish peroxidase conjugate. The signal was detected with 3,3′-diaminobenzidine substrate, and the sections were counterstained with methyl green.

Immunohistochemistry was performed to detect the impact of dopamine instillation on dopaminergic amacrine cells [39]. The primary antibodies, mouse anti-tyrosine hydroxylase (monoclonal, MA1100, 1:100; Boster Bio, Pleasanton, CA, USA) and rabbit anti-CALB2 (polyclonal, PAB7957, 1:100; Abnova, Taipei, Taiwan), were incubated overnight at 4 °C. Subsequently, an incubation was conducted at room temperature for one hour with the secondary antibody, Alexa-488 donkey anti-mouse or Alexa-594 donkey anti-rabbit IgG (1:200). DAPI stain (F6057; Fluoroshield, Sigma-Aldrich, Livonia, MI, USA) was used for nuclear staining.

### 2.6. Statistical Analysis

The data were subjected to statistical analysis using the SPSS software, version 22.0 (SPSS Inc., Chicago, IL, USA). The Wilcoxon signed rank test was used to compare the difference in axial lengths between the FD eyes with topical instillation and the contralateral untreated eyes. The Mann–Whitney U test was used for comparisons between the dopamine group and the control groups. *p* values of less than 0.05 were considered statistically significant. Data are presented as mean ± standard deviation unless noted otherwise.

## 3. Results

### 3.1. Efficacy of Topical Dopamine in Rabbits

**Figure 2:**
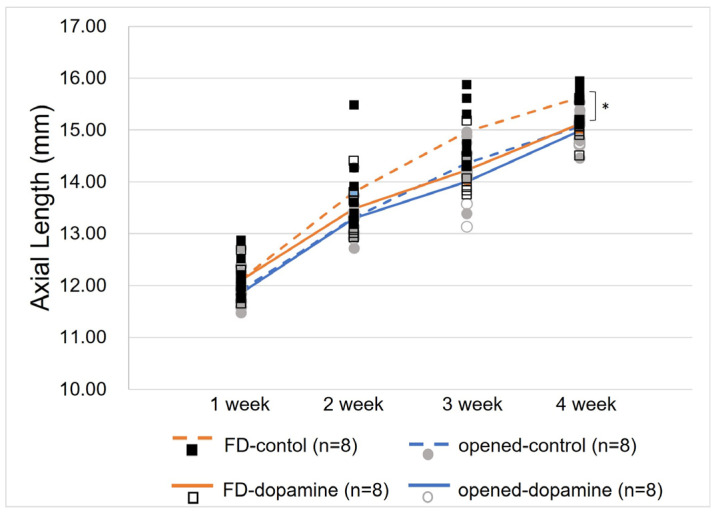
Change in axial lengths of both eyes in the control and dopamine groups. After 4 weeks, a statistically significant difference in axial lengths was observed between the form-deprivation eyes in the dopamine group and the control group (15.07 ± 0.34 mm, 15.63 ± 0.33 mm, respectively, *p* = 0.015, Mann–Whitney U test). In contrast, no significant difference was observed between the open contralateral eyes of the two groups (14.98 ± 0.29 mm, 15.12 ± 0.36 mm, respectively, *p* = 0.328, Mann–Whitney U test). * *p* value < 0.05 by Mann–Whitney U test.

The axial lengths of each group by age are described in Table 1. A comparison of the dopamine group and the control group revealed no significant difference in the axial length of the contralateral opened eye between the two groups (14.98 ± 0.29 mm, 15.07 ± 0.34 mm, respectively, *p* = 0.328). However, the axial length of the FD eye was found to be significantly shorter in the dopamine group than in the control group (15.12 ± 0.36 mm, 15.63 ± 0.33 mm, respectively, *p* = 0.015) (Figure 2).

**Figure 3:**
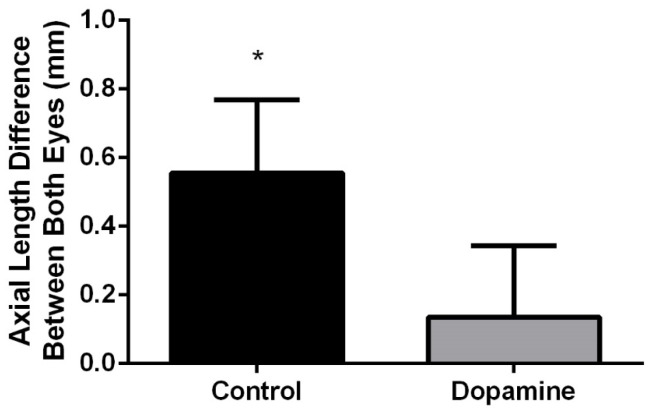
Axial length differences between both eyes after treatment. Significant axial length differences between both eyes were observed in the control group (*p* = 0.012, Wilcoxon signed rank test), whereas the dopamine group showed no significant difference in both eyes, indicating that topical dopamine application effectively inhibited myopia growth in the dopamine group (*p* = 0.123, Wilcoxon signed rank test). * *p* value < 0.05 by Wilcoxon signed rank test.

At the end of the 4-week treatment period, the axial length of the diffuser attached FD eye in the control group was significantly longer than that of the contralateral opened eye (inter-eye difference, 0.56 ± 0.21 mm, *p* = 0.012). In contrast, there was no statistically significant difference between the axial length of the FD eye and the contralateral eye in the dopamine group. (inter-eye difference 0.14 ± 0.21 mm, *p* = 0.123) (Figure 3).

### 3.2. Safety of Topical Dopamine in Rabbits

**Figure 4:**
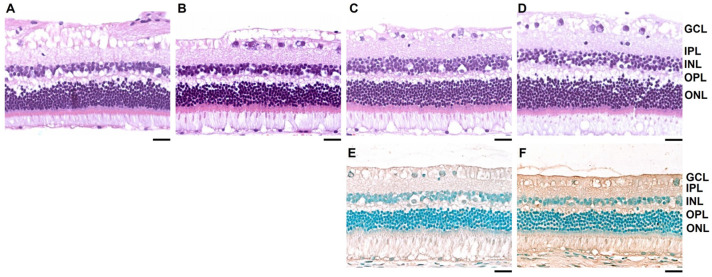
Retinal histology after 4 weeks of treatment. Rabbit retinae were stained with hematoxylin and eosin. (**A**–**D**) (**A**) Opened contralateral untreated eye in the control group. (**B**) Form-deprivation (FD) eye with normal saline instillation in the control group. (**C**) Opened contralateral untreated eye in the dopamine group. (**D**) FD eye with topical dopamine instillation in the dopamine group. (**E**) Terminal deoxynucleotidyl transferase dUTP nick end labeling (TUNEL) assay of the untreated contralateral eye of the dopamine group. (**F**) TUNEL assay of the FD eye with topical dopamine instillation. No structural changes or cell apoptosis are observed in the retina (**A**–**F**). Scale bar: 200 μm. GCL: ganglion cell layer; IPL: inner plexiform layer; INL: inner nuclear layer; OPL: outer plexiform layer; ONL: outer nuclear layer.

The slit lamp examination revealed no abnormalities in the FD eye that had been instilled with dopamine for four weeks and in the contralateral open eye. Histological analysis using H&E and TUNEL assays of the FD eyes showed no retinal structural changes or apoptosis (Figure 4).

**Figure 5:**
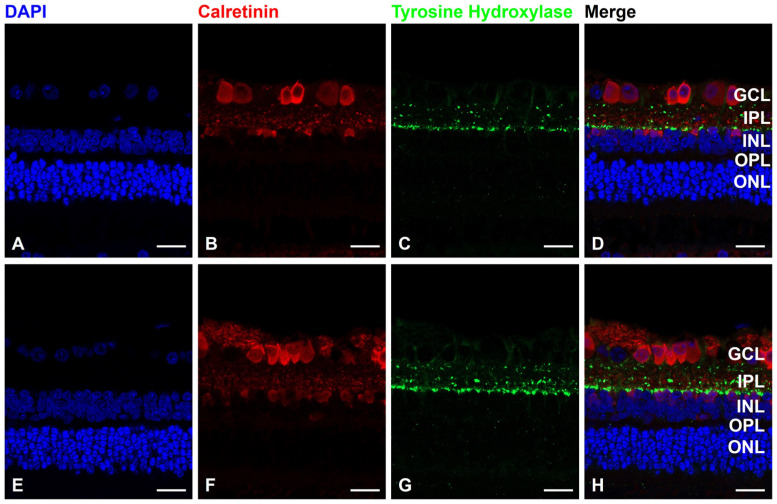
Form-deprivation (FD) with normal saline treatment, (**A**–**D**) FD with dopamine instillation. (**E**–**H**) Immunofluorescence staining with 4′,6-diamidino-2-phenylindole (DAPI) for nuclei (blue), calretinin antibody for amacrine cells (red) and tyrosine hydroxylase antibody for dopaminergic amacrine cells (green). Compared with the control group, 4 weeks of dopamine instillation showed no change in dopaminergic amacrine cells. Scale bar: 20 μm. GCL: ganglion cell layer; IPL: inner plexiform layer; INL: inner nuclear layer; OPL: outer plexiform layer; ONL: outer nuclear layer.

Immunofluorescence staining with a tyrosine hydroxylase antibody indicated that dopaminergic amacrine cells in the FD eye and the contralateral eye exhibited no changes (Figure 5).

## 4. Discussion

This study showed that a light diffuser could be used to effectively create an experimental model of FD myopia in rabbits. Topical dopamine instillation could effectively inhibit axial length elongation. The eyes of the control group, which had been attached to a diffuser for four weeks, exhibited a significantly greater axial length compared to the contralateral eye. In contrast, the axial length of the diffuser-attached eyes of the dopamine group and the contralateral eye did not differ significantly. Furthermore, the administration of dopamine for a period of four weeks did not result in any observable damage to the cornea, conjunctiva, or lens. Additionally, it did not induce any structural alterations, cell death, or changes in dopaminergic amacrine cells within the retina.

Although the pathophysiology of myopia is still poorly understood, the leading hypothesis for the regulation of myopia is that dopamine is a key neurotransmitter [14,15]. Since Stone et al. [8] demonstrated that dopamine levels are reduced in FD myopia, it has been shown that dopamine or levodopa injected directly into the eye can prevent FD myopia in rabbits [32] and guinea pigs [31]. In addition, the dopamine receptor agonist, such as apomorphine, has also been shown to prevent FD myopia in a variety of species, including chickens [40], guinea pigs [19], monkeys [9] and mice [41]. Recent studies, not only animal studies but also clinical evidence, showing a strong correlation between time spent outdoors and light intensity with myopia suppression, support the hypothesis that retinal dopamine, which is synthesized and released by light stimulation, plays a role in myopia control [22,42,43,44,45].

However, inconsistent findings across species regarding the effects of dopamine have occasionally arisen. For example, in chicks [46] and tree shrews [47], dopamine appeared to inhibit experimental myopia through a D2-like receptor mechanism, whereas in mice [48], protection appears to occur through a D1-like receptor mechanism. These are thought to be due to species-specific anatomical differences in the distribution of dopamine receptors and photoreceptors, which suggests that studies in a variety of species are needed to understand the role of dopamine [49]. Rabbits, which were used in our research, are a commonly employed animal in ophthalmic research due to their phylogenetic proximity to humans compared to chicks or rodents, and their cost-effectiveness compared to primates. However, the use of rabbit kits for myopia research presents several challenges. As demonstrated by Gao’s research [32], the survival rate of artificially bred rabbits is extremely low. Therefore, it is necessary to raise them with their mother for the first few weeks of their lives. However, mother rabbits are highly sensitive and will readily abandon their young immediately after birth if the breeding room is not tranquil and/or there are other rabbits present, even in a separate cage [50]. It is therefore essential to provide the mother rabbit with adequate food, snacks, enrichment toys, and hiding places, and to construct an independent birthing cage in a very quiet environment [51].

A more clinically relevant and preferable approach for longer-term pharmacological treatment is the administration of drugs via topical eye drops. Repeated ocular injection is an invasive procedure with the risk of infection, which makes it challenging to apply in clinical practice, particularly in young children with myopia progression. Iuvone et al. [16] demonstrated that local application of apomorphine reduced the development of FD myopia in infant rhesus monkeys, while Thomson et al. [33,34] showed that topical levodopa application may have potential for myopia control in chick. Our study was the first to test topical dopamine in rabbits and demonstrated that topical application of dopamine has the therapeutic potential and safety for axial length modulation.

Our study has certain limitations. First, the experiment was conducted with a small number of rabbits. However, in accordance with animal ethics, which prohibits unnecessary overuse of experimental animals, the minimum number possible for statistical analysis was enrolled. Second, we measured axial length only. Because retinoscopic refraction in small animal eyes tends to be overly hyperopic, individualized custom refraction is recommended for studies where refractive state is critical [52,53]. However, we were not able to obtain a custom built auto-refractor, such as an eccentric infrared photorefractor designed for rabbit kits, because it does not exist [54,55]. Third, the observation period was short as it was terminated at the point when statistically significant differences in axial length were observed. Thus, long-term intraocular safety of topical dopamine should be investigated further. Finally, we only performed safety evaluation using the slit lamp and in vitro histology. Future studies should include in vivo assessment such as optical coherence tomography and electroretinography.

## 5. Conclusions

**Figure 6:**
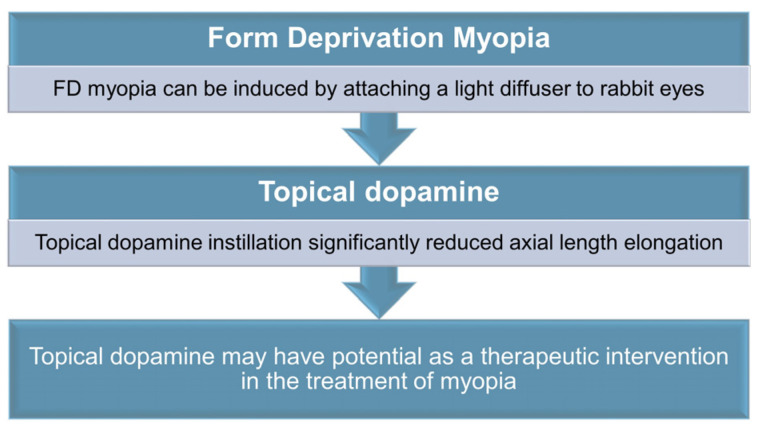
Conclusion figure to highlight the importance of the outcomes.

In conclusion, FD myopia can be induced by attaching a light diffuser to rabbit eyes. Furthermore, topical administration of dopamine eyedrops inhibits the development of experimental FD myopia without causing ocular toxicity. These findings indicate that topical dopamine may have potential as a therapeutic intervention for myopia (Figure 6).

## Figures and Tables

**Table 1:** The axial length (mm) for each weekly age.

Group		Week 1		Week 2		Week 3		Week 4	
		Mean ± SD	Median	Mean ± SD	Median	Mean ± SD	Median	Mean ± SD	Median
Dopamine	FD	12.10 ± 0.33	12.10	13.49 ± 0.51	13.44	14.23 ± 0.47	14.18	15.12 ± 0.36	15.10
	Open	11.86 ± 0.24	11.89	13.30 ± 0.25	13.30	14.01 ± 0.45	14.19	14.98 ± 0.29	15.03
Control	FD	12.12 ± 0.39	12.00	13.81 ± 0.77	13.50	14.97 ± 0.55	14.72	15.63 ± 0.33	15.73
	Open	11.91 ± 0.41	11.86	13.31 ± 0.48	13.30	14.36 ± 0.52	14.01	15.07 ± 0.34	14.98

SD: standard deviation, FD: Form deprivation.

## Data Availability

The datasets used and/or analyzed during the current study are available from the corresponding author on reasonable request. For data requests, please contact 98614@snubh.org.
